# Development of a simple polymer-based sensor for detection of the Pirimicarb pesticide

**DOI:** 10.1038/s41598-024-60748-6

**Published:** 2024-05-04

**Authors:** Zahra Saadatidizaji, Negin Sohrabi, Reza Mohammadi

**Affiliations:** 1 Polymer Research Laboratory, Department of Organic and Biochemistry, Faculty of Chemistry, University of Tabriz, Tabriz, Iran; 2 Department of Biosystem Engineering, Faculty of Agriculture, University of Tabriz, Tabriz, Iran

**Keywords:** Molecularly imprinted polymer, Pirimicarb, Graphene quantum dots, Fluorescent sensor, Adsorption, Environmental sciences, Chemistry

## Abstract

In this study, a sensitive and selective fluorescent chemosensor was developed for the determination of pirimicarb pesticide by adopting the surface molecular imprinting approach. The magnetic molecularly imprinted polymer (MIP) nanocomposite was prepared using pirimicarb as the template molecule, CuFe_2_O_4_ nanoparticles, and graphene quantum dots as a fluorophore (MIP-CuFe_2_O_4_/GQDs). It was then characterized using X-ray diffraction (XRD) technique, Fourier transforms infrared (FT-IR) spectroscopy, scanning electron microscope (SEM), and transmission electron microscopy (TEM). The response surface methodology (RSM) was also employed to optimize and estimate the effective parameters of pirimicarb adsorption by this polymer. According to the experimental results, the average particle size and imprinting factor (IF) of this polymer are 53.61 nm and 2.48, respectively. Moreover, this polymer has an excellent ability to adsorb pirimicarb with a removal percentage of 99.92 at pH = 7.54, initial pirimicarb concentration = 10.17 mg/L, polymer dosage = 840 mg/L, and contact time = 6.15 min. The detection of pirimicarb was performed by fluorescence spectroscopy at a concentration range of 0–50 mg/L, and a sensitivity of 15.808 a.u/mg and a limit of detection of 1.79 mg/L were obtained. Real samples with RSD less than 2 were measured using this chemosensor. Besides, the proposed chemosensor demonstrated remarkable selectivity by checking some other insecticides with similar and different molecular structures to pirimicarb, such as diazinon, deltamethrin, and chlorpyrifos.

## Introduction

Pesticides have become more widely used in recent years for the control and eradication of pests owing to high agricultural product demand^1^. Pirimicarb[2-(dimethylamino)-5, 6-dimethyl-4-pyrimidinyl dimethylcarbamate] is a pesticide from the carbamate insecticide family that has been widely used to control aphids on a variety of agricultural and horticultural crops because of their diverse range of biological function^2,3^. Despite all this, it is very poisonous, and excessive use would undoubtedly contaminate the environment. Moreover, carbamate residues in the food chain may suppress acetylcholinesterase action at the synapse, resulting in nervous system malfunction. As a result, extremely sensitive and selective analytical techniques for monitoring pesticides should be developed to offer an early warning and prevent their residues from growing in the environment^4^. As regards this purpose, chromatographic techniques, in conjunction with a variety of detectors, such as gas chromatography (GC), high-performance liquid chromatography (HPLC), and mass spectrometry (MS) have been the most extensively used in the past few decades^5,6^. It is worth noting that although these techniques provide high accuracy, sensitivity, and reproducibility, they have a lot of limitations. For instance, heavy and expensive equipment, time-consuming sample preparation, tedious procedures, being inappropriate for on-site and real-time monitoring, the usage of considerable amounts of organic solvents, and so on. Recently, sensors with excellent selectivity and sensitivity, and also simple, rapid, low-cost, high-throughput, and suitable for on-site detection, have evolved into a flexible sensing platform for pesticide remnant analysis^7,8^. A sensor usually consists of a transducer and a processor in order to provide selective and quantitative analytical information using a biological/chemical/electronical recognition element^9^. The most commonly reported types of sensors are the optical sensors, which are established by the interaction of the optical field with a recognition element. Among them, fluorescence-based optical sensors have been considerably studied for medical diagnostic, environmental, and food quality assessment due to their excellent selectivity, sensitivity, and fast reaction time^10,11^. Fluorescence is an optical phenomenon that involves labeling an analyte or molecules for detection. Various fluorescent dyes, such as QDs, dyes, and fluorescent proteins, are utilized in these sensors^12,13^. Regarding the construction of a sensor, artificially manufactured molecularly imprinted polymers (MIPs) as a preferable option for recognition elements provide the selective detection and separation of target molecules^8^. Molecularly imprinted polymers are simple to prepare, stable, and inexpensive. Also they can be generated in large amounts with excellent reusability. Imprinted polymers are more physically robust, resistant to high temperatures and pressures, and inert to acids, bases, metal ions, and organic solvents than biological systems like proteins and nucleic acids. Therefore, they have attracted a great deal of attention in various fields, such as solid phase extraction, chiral separation, simulating antibodies, drug delivery, treatment of wastewater, and chemical sensors. The main advantages of MIPs are their exceptional affinity and selectivity for the analyte molecule. However, some shortcomings develop during the imprinting of cavities, which makes it possible for several additional molecules to enter the imprinting cavities and interfere with the small molecules. Moreover, water molecules can compete with the template, weakening or eliminating non-covalent interactions (such as electrostatic, hydrogen, and van der Waals bonds) between the template and functional monomer. So, further development of the imprinting mechanism of recognition at the molecular level and the imprinting process to generate imprinted cavities with high stable structure, high accuracy, and a high degree of order are required to improve the selectivity and avoid interfering with capacity. Also, to improve template and functional monomer association in water, hydrophobic, ionic, or metal co-ordination interactions have been demonstrated to be highly promising^14–16^.

The approach is primarily based on molecular imprinting, which is a type of polymerization that occurs around targets known as templates and forms unique holes in the polymeric matrix. Following the elimination of the template, three-dimensional specific holes are created^17,18^. In order to increase the number of recognition areas on the polymer surface, improve the system's sensitivity and detectability, and reduce its response time, the appropriate method is to provide an extensive surface area, either by combining polymer films with nanomaterials or by producing Nano-sized MIPs. The nanostructured imprinted materials have high surface-to-volume ratios, which improve both analyte accessibility and kinetics^19^.

Graphene quantum dots (GQDs) have received a great deal of interest in recent years. They offer several extraordinary features, including exceptional electrical conductivity, significant physical and chemical properties, excellent optical qualities, high fluorescence quantum yields, and low toxicity^20,21^. Meanwhile, CuFe_2_O_4_ magnetic nanoparticles have attracted interest as an alternative catalyst due to their specific chemical and physical features, which can be employed in various fields, especially sensing^22,23^. One of the advantages of the CuFe_2_O_4_ NPs in comparison with monometallic nanoparticles such as Fe_2_O_3_ NPs are that they are bimetallic and often have a higher surface-to-volume ratio, which improves surface polymerization and, as a result, increases binding sites.

**Scheme 1:**
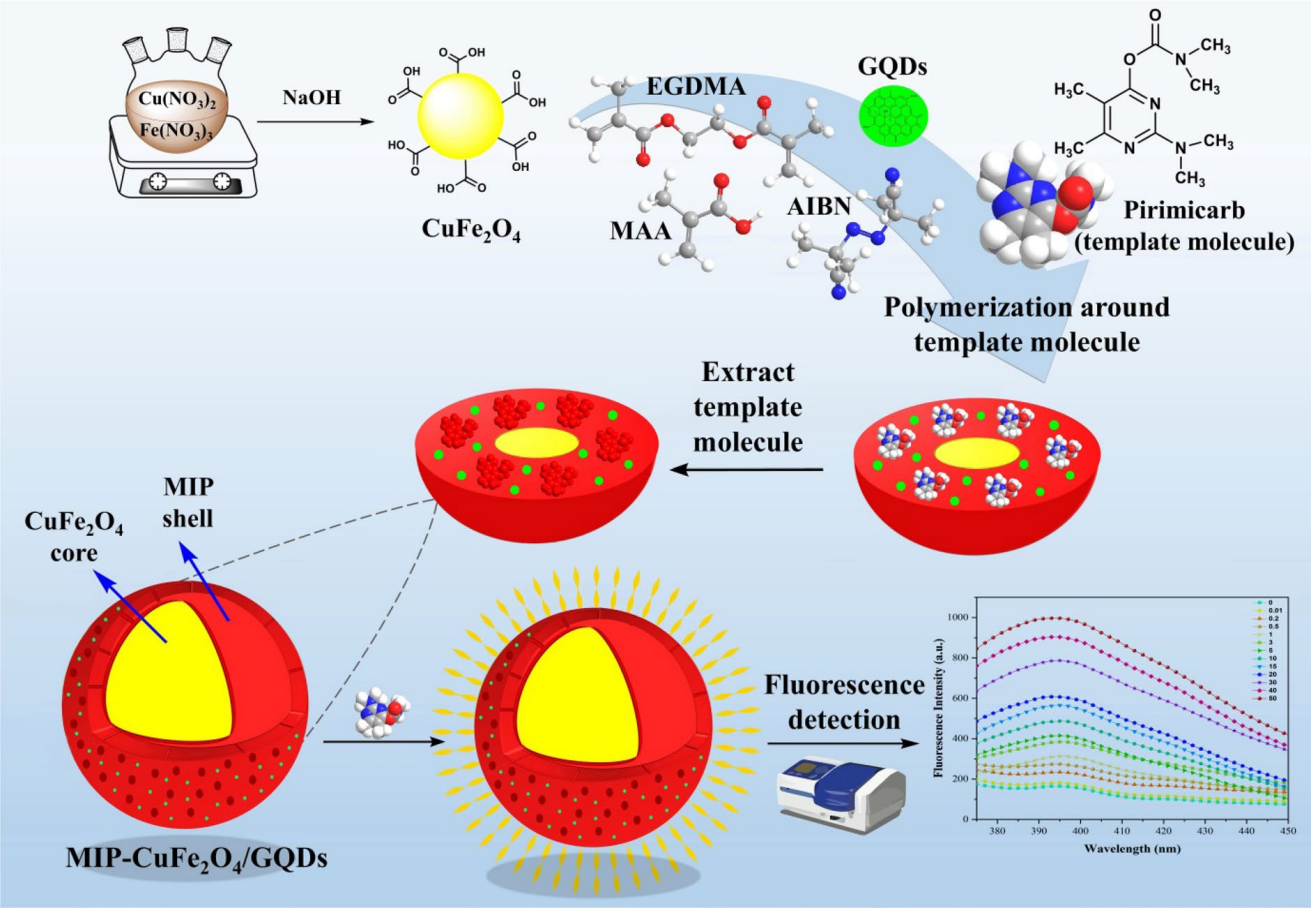
Schematic illustration of the fluorescent chemosensor for determination of pirimicarb based on MIP-CuFe_2_O_4_/GQDs.

Therefore, in this study, the pirimicarb was measured by synthesizing and characterizing a magnetic molecularly imprinted polymer (MIP-CuFe_2_O_4_/GQDs) and using fluorescence spectroscopy (Scheme 1). For preparing of the nanocomposite, initially, CuFe_2_O_4_ nanoparticles and GQDs were synthesized separately and then utilized for producing the MIP. Various solutions with distinct concentrations of pirimicarb and a specific amount of MIP were prepared, and adsorption efficiency was measured with fluorescence spectrophotometry. As a result, the measured fluorescence intensity had a positive correlation with the concentration of pirimicarb, and the developed chemosensor exhibited high selectivity and sensitivity to the pirimicarb.

## Material and methods

### Materials

Cu(NO_3_)_2_ (99.99%), Fe(NO_3_)_3_ (98%), NaOH (98%) solution, acetone (99.5%), and chloroform (99%) were obtained from Merck company and utilized without further purification. For the synthesis of graphene quantum dots, citric acid monohydrate (Merck, 98%) was used. Pirimicarb was ordered from Mahan Co. (Tabriz, Iran). Ethylene glycol dimethacrylate (EGDMA) (97.5%) and Methacrylic acid (MAA) (99.6%) were acquired from Sigma-Aldrich, and Azobisisobutyronitrile (AIBN) (96%) was purchased from Merck.

### Apparatus

The characteristics and surface properties of the pirimicarb molecularly imprinted polymer (MIP-CuFe_2_O_4_/GQDs) have been evaluated using various analyses. In this study, Fourier transform infrared (FT-IR) spectroscopy (Tensor 27, Bruker) was performed to identify the functional groups and their interactions with pirimicarb pesticide. The crystalline structures of prepared materials were analyzed by X-ray diffraction (XRD) technique (Tongda, model TD-3700, china). Moreover, the scanning electron microscope (SEM) was utilized to investigate the surface topography and composition of the aforementioned nanocomposite (TESCAN MIRA3 FEG-SEM). Transmission Electron Microscopy (TEM) images of CuFe_2_O_4_ and MIP-CuFe_2_O_4_/GQDs was recorded using Philips EM 208S. The residual pirimicarb concentrations at any step were determined with a UV–VIS spectrophotometer (Specord 250, Analytik Jena), and a fluorescence spectrophotometer (LS45, PerkinElmer) was applied for the sensing process.

### Preparation of CuFe_2_O_4_

For a typical synthesis of CuFe_2_O_4_ nanoparticles, initially Cu(NO_3_)_2_ (1870 mg) and Fe(NO_3_)_3_ (4820 mg) were dissolved in deionized water (100 mL) in 1:2 ratios, followed by 1 h of stirring at 90 °C temperature under the inert gas (N_2_). Then, NaOH solution (30 ml, 5 M) was added dropwise to the resulting solution under stirring. After 1 h, the obtained precipitate was separated using an external magnet (3T) and washed several times with deionized water, and dried in the oven at 90 °C for 24 h.

### Preparation of GQDs

The graphene quantum dots were synthesized through the bottom-up technique by pyrolysis of citric acid following the protocols of the previous report^24^. Briefly, 2000 mg of citric acid monohydrate was melted at 200 °C for 45 min in the oven and then added dropwise to 100 ml of NaOH solution with a 10 mg/ml concentration. Then, the resultant mixture was neutralized with 0.1 M NaOH after 10 min.

### Synthesis procedure of polymers

In order to prepare the pirimicarb imprinted polymer, first 50 mg of pirimicarb was dissolved in chloroform (30 ml). Afterward, 100 mg of CuFe_2_O_4_ nanoparticles and 100 mg of GQDs were dispersed in it, respectively. (10 min, by ultrasonic dispersion) Then 135 µL of MAA as a functional monomer, EGDMA as a cross-linking monomer (755 µL), (the ratio of template: functional monomer: cross linker = 1:8:20) and AIBN as an initiator (16 mg) were added to the obtained solution, respectively. Meanwhile, degassing was carried out before the addition of AIBN for 3 min and about 5 min after its addition. The final product (915 mg) was obtained after 19 h and at a temperature of 50 °C. Subsequently, the resultant magnetic polymer was extracted from the mixture by an external magnet. Then, it was washed with a solution including 90% acetone and 10% acetic acid by the Soxhlet method to remove the template molecule (pirimicarb) from the produced imprinted polymer construction. Non-imprinted polymer (NIP) was prepared (668 mg) in the same method as the MIP nanocomposite but without the addition of the pirimicarb. In this study, the polymer was prepared using MAA as the functional monomer and EDMA as the cross linking monomer. Hydrogen bonds are expected to be formed between the nitrogen atoms of pirimicarb and MAA as a key interaction for binding-site construction^3,25–27^. Also, in this study, we have synthesized several polymers based on different ratio of template, functional monomer, and cross linker such as 1:4:20, 1:8:20, and 1:16:20. Finally, we found mentioned ratio (1:8:20) as the best value.

### Assessment of effective adsorption parameters

To study the adsorption process of pirimicarb by MIP-CuFe_2_O_4_/GQDs nanocomposite, pirimicarb solutions of various values, including 10, 20, 30, 40, and 50 mg/L, were provided. pH was adjusted using 0.1 M NaOH or HCl (3 to 11). Then, specific dosages of MIP-CuFe_2_O_4_/GQDs (250 to 1250 mg/L) were added to the solutions, and the resulting compounds were stirred for 5 to 25 min. In all samples, after the pirimicarb adsorption mechanism, the MIP-CuFe_2_O_4_/GQDs were removed from the solution with an external magnet, and the concentration of the remaining pesticide in the solution was determined by a UV-1700 spectrophotometer at 313 nm. According to the results, the adsorption yield of pirimicarb by the nanocomposite and the adsorption capacity were calculated by applying Eqs. (1) and (2), respectively^28^.

1 $$R\% = \left( {\frac{{{C_i} - {C_e}}}{{C_i}}} \right) \times 100$$

2 $${q_e} = \left( {{C_i} - {C_e}} \right) \times \frac{V}{m}$$

In the equations above, R is the adsorption percentage, q_e_ is the amount of adsorption at equilibrium (g/kg), and C_i_ and C_e_ are the initial and equilibrium concentrations of pirimicarb (mg/L), respectively. V is the liquid volume (L), and m is the amount of polymer (g).

### Response surface methodology (RSM) experiments

To optimize pirimicarb adsorption by MIP-CuFe_2_O_4_/GQDs nanocomposite, a four-factor based model was designed using RSM (CCD) with Design Expert 11 software. For four independent variables (k), namely pH, time, initial pirimicarb concentration, and polymer quantity, the 28 (2^k^ + 2k + 4 = 28) experiments suggested by this model were carried out in this study.

Table 1 shows the ranges, levels, and design matrix of chosen parameters. In addition, the model suggested by response surface methodology utilizing analysis of variance was evaluated. This model contained predictor variables and demonstrated the correlation between independent and dependent variables. Significant and non-significant impacts of independent variables were investigated using an analysis of variance and a P-value. The model predictions were then compared to the experimental results and the correlation coefficient (R^2^) was calculated. Further relevant statistics for analyzing model performance and efficiency, such as standard deviation (SD), coefficient of variation (CV %), desirability function (DF), and Adequate precision (AP), were also investigated.

**Table 1:** Independent variables and levels of RSM.

Independent variables	Coded symbol	Unit	Center level			Axial level	
			Low (− 1)	Center (0)	High (+ 1)	Minimum (− α)	Maximum (+ α)
pH	X_1_	–	5	7	9	3	11
Time	X_2_	min	10	15	20	5	25
Concentration	X_3_	mg/L	20	30	40	10	50
Dosage	X_4_	g/L	0.5	0.75	1	0.25	1.25

### Fluorescence measurements and sensitivity determination

To evaluate the fluorescence performance, several solutions were prepared at a pirimicarb concentration range of 0 to 50 mg/L (10 ml) and optimal pH. Then 1 mg of MIP-CuFe_2_O_4_/GQDs was dispersed (5 min) in all of them. The fluorescence intensity of each sample with an excitation wavelength of 370 nm was obtained in 10 s with a fluorescence spectrophotometer at 375–450 nm wavelengths. Then, the limit of detection (LOD) and limit of quantitation (LOQ) were calculated from the calibration line at low concentrations by the following equations (Eqs. (3) and (4))^29^. In order to check the ability of the sensor, real river water samples (Table S3) with the addition several concentration of pirimicarb (0 to 50 mg/L) were investigated. Then, based on the measured fluorescence intensity and the sensor response equation, the concentration of pirimicarb was measured. Relative Standard Deviation (RSD) for this measurement was calculated and then the accuracy of this method based on RSD was investigated.

3 $$LOD = 3.3{{S_a} \mathord{\left/ {\vphantom {{S_a} b}} \right. \kern-0pt} b}$$

4 $$LOQ = 10{{S_a} \mathord{\left/ {\vphantom {{S_a} b}} \right. \kern-0pt} b}$$

In these equations, S_a_ and b are the standard deviation of the intercept and the slope of the regression line, respectively. The response equation and linear model (approximately) of the sensor were also obtained based on the results of this section. Then, the sensitivity of the sensor was determined from the slope of the approximately linear model.

### Selectivity and binding study

To investigate the specificity of the NIP-CuFe_2_O_4_/GQDs and MIP-CuFe_2_O_4_/GQDs nanocomposites, the binary solutions of three other different pesticides (namely Diazinon, Deltamethrin, and Chlorpyrifos) with pirimicarb (10 mg/L) were prepared. Then, 20 mg dosages of nanocomposites were added to the solutions separately, and after 1 h of stirring, they were removed from the solutions with an external magnet. The adsorption experiments were performed under identical experimental conditions for all six solutions, and the concentration of residual pesticides in solutions was measured by spectrophotometry. According to the results, the distribution coefficients of each pesticide were obtained using Eq. (5).

5 $${K_d} = \frac{{q_e}}{{C_e}}$$

K_d_ is the pesticide distribution proportion (L/g) between the adsorbent and the aqueous phase, q_e_ is the quantity of adsorption at equilibrium (g/kg), and C_e_ denotes the pesticide's equilibrium concentration (mg/L).

The selectivity coefficient of each adsorbent (K) for pirimicarb with regard to the competitive pesticides were calculated by Eq. (7). The proportion of K_MIP_ to K_NIP_ (Eq. (7)) was then used to calculate the relative selectivity coefficient (K′)^30^.

6 $$K = \frac{{{K_d}\left( {{\text{pirimicarb}}} \right)}}{{{K_d}\left( {\text{competitive pesticide}} \right)}}$$

7 $$K^{\prime} = \frac{{{K_{MIP}}}}{{{K_{NIP}}}}$$

Moreover, binding tests and Scatchard analysis were applied to assess the binding site and calculate the imprinting factor of the MIP. Pirimicarb binding experiments were carried out with a 10 mg dosage of each nanocomposite at a pirimicarb concentration range of 10 mg/L to 50 mg/L under optimal conditions. The amount of pirimicarb adsorbed on the polymers was calculated to be applied in the Scatchard equation (Eq. (8)) through measuring the ultimate and initial concentration of pirimicarb with UV spectrophotometry.

8 $$\frac{q}{{C_e}} = \frac{{{q_{\max }} - q}}{{K_D}}$$

In this equation, q is the binding/adsorption capacity of nanocomposites, and C_e_ is the equilibrium concentration of pirimicarb. The K_D_ and q_max_ are defined as the equilibrium association constant and apparent capacity, which were obtained from the slope and intercept of the line graph wherein q/C_e_ is plotted versus q, respectively^31^.

Following that, by computing the ratio of the apparent capacity of MIP to NIP, the imprinting factor (IF) was obtained^32^.

### Reusability

The reusability of adsorbents is an essential and effective parameter for sensor material. The adsorption process was performed in optimal conditions, and desorption was done at pH 1 to 13 of Britton–Robinson buffer. The best solution was selected based on the desorption percentage (Calculated from Eq. (9))^33^. Afterward, the adsorption test was performed. Then the adsorbent was separated from the solution and dried, and a desorption test was performed. This process was repeated for up to 15 cycles.

9 $$\%{\text{ of Desorption}} = \left( {\frac{{\text{amount of pirimicarb desorbed}}}{{\text{amount of pirimicarb adsorbed}}}} \right) \times 100$$

## Results and discussion

### Characterization of nanocomposite and polymers

**Figure 1:**
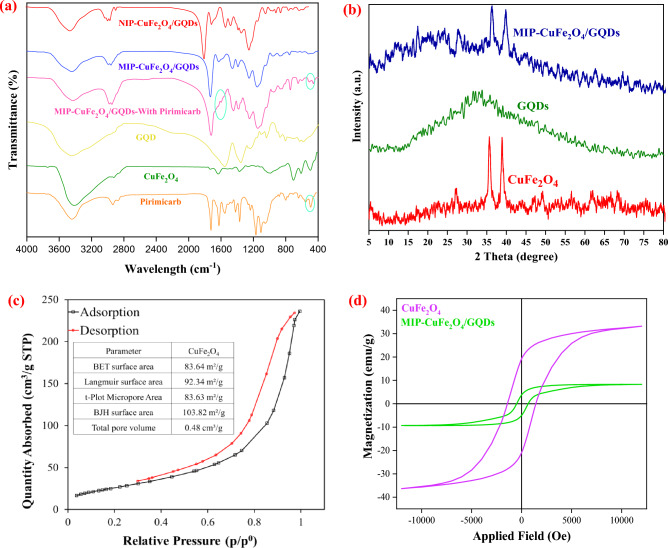
(**a**) FT-IR spectra of the Pirimicarb, CuFe_2_O_4_, GQD, NIP-CuFe_2_O_4_/GQDs, and MIP-CuFe_2_O_4_/GQDs nanocomposites (before and after removing the pirimicarb), (**b**) XRD pattern of CuFe_2_O_4_, GQDs, and MIP-CuFe_2_O_4_/GQDs, (**c**) BET analysis for CuFe_2_O_4_ NPs, (**d**) VSM analysis for CuFe_2_O_4_ and MIP-CuFe_2_O_4_/GQDs.

The FT-IR spectra of CuFe_2_O_4_ nanoparticles, GQD, NIP-CuFe_2_O_4_/GQDs, and MIP-CuFe_2_O_4_/GQDs nanocomposites are shown in Fig. 1a. According to the spectrum of CuFe_2_O_4_, there are two main absorption bands at about 493 and 703 cm^−1^, which are attributed to stretching vibrations of the octahedral and tetrahedral sites in spinels (Cu–O and Fe–O bonds), respectively^34^. Moreover, the two peaks at 1633 and 3416 cm^−1^ represent the adsorption peak of C=O and stretching and bending vibrations of O–H groups. The FT-IR spectrum of GQDs was shown in Fig. 1a, there is a broad peak at 3453 cm^−1^ is related to –OH and –NH groups stretching vibration. Also, the well-defined peak at 1585 cm^−1^ is assigned to bending vibrations of C=C group. The peaks at 1389 cm^−1^, and 1077 cm^−1^ were related to the C−O (carboxy), and C−O (alkoxy) functional groups, respectively^35^. Besides, the spectra of the MIP-CuFe_2_O_4_/GQDs indicate three additional significant absorption peaks at 2989, 1731, and 1156 cm^−1^, which respectively represent the presence of the methylene group in both MAA and EGDMA, stretching vibration of C=O and C–O bonds^36–39^. Also, According to the FT-IR spectrum of the pirimicarb, the peaks of this pesticide overlap with NIP and MIP nanocomposites. There is only one peak in the region of 450 cm^−1^, which can also be seen in the polymer before removing the template from it. The peak in the region of 1600 cm^−1^ is also present in the spectrum related to polymer, but its intensity has been changed. Basically, in the synthesis of molecular imprinted polymer, the amount of template molecule is less and it is quite probable that the intensity of its peaks is low. In addition, the peak observed in the region of 2900 cm^−1^ in the MIP-CuFe_2_O_4_/GQDs-with pirimicarb is high intensive than the NIP-CuFe_2_O_4_/GQDs, which can be caused by the presence of pirimicarb in the structure of the MIP with pirimicarb. Also it has been decreased in intensity after washing in MIP-CuFe_2_O_4_/GQDs^27^.

**Figure 2:**
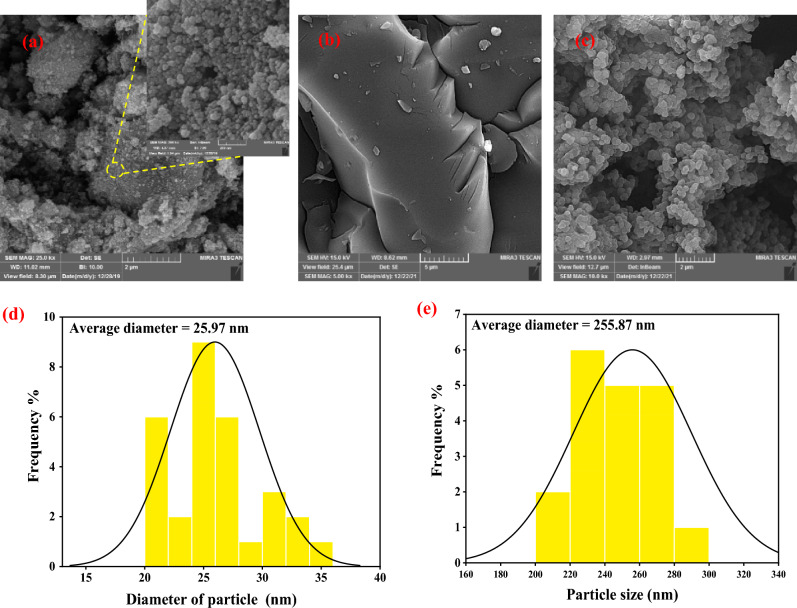
(**a**) SEM image of CuFe_2_O_4_ NPs, (**b**) SEM image of GQDs, (**c**) SEM images of the MIP-CuFe_2_O_4_/GQDs nanocomposite, (**d**) Particle size distribution of CuFe_2_O_4_ NPs, (**e**) Particle size distribution of MIP-CuFe_2_O_4_/GQDs.

Figure 1b presents the XRD patterns of the CuFe_2_O_4_, GQDs, and MIP-CuFe_2_O_4_/GQDs nanocomposite. As can be observed, the XRD pattern of the CuFe_2_O_4_ indicates eight definite peaks in the range of 2θ, which are indexed to (111), (220), (311), (222), (400), (511), (440), and (533) crystallographic planes of CuFe_2_O_4_, respectively^40,41^. These peaks can also be seen in the pattern of the MIP-CuFe_2_O_4_/GQDs with a slight shift and discrepancy in the intensity, which confirms that the crystalline phase of CuFe_2_O_4_ nanoparticles is not altered by MIP synthesis^42^. Besides, in the XRD pattern of GQDs, a broad peak appeared at around 2θ = 33.75 concerning the (002) plane^43^, and its slight effect on the XRD pattern of the nanocomposite can be observed. For both CuFe_2_O_4_ nanoparticles and MIP-CuFe_2_O_4_/GQDs nanocomposite, the distance between the crystal plates was determined by using the Bragg law, and the size of the crystals was calculated using the Debye–Scherrer relationship. According to the results, the crystal sizes for them are 26.5 and 13.2 nm, respectively, and the distance between the crystal plates is equal to 0.253 nm.

The result of BET analysis is shown in Fig. 1c. As can be seen, the surface area of CuFe_2_O_4_ NPs in BET isotherm is equal to 83.64 m^2^/g. The surface-to-volume ratio of CuFe_2_O_4_ NPs is very large and more suitable compared with other nanoparticles such as iron nanoparticles. The BET surface area of Fe_2_O_3_ NPs^44^ and CuFe_2_O_4_ NPs^45^ approximately were equal to 30 m^2^/g and 63 m^2^/g, respectively.

Vibrating sample magnetometer (VSM) analysis was used to investigate and determine the magnetic properties of CuFe_2_O_4_ nanoparticles and MIP-CuFe_2_O_4_/GQDs nanocomposite (Fig. 1d). The results showed that the CuFe_2_O_4_ nanoparticles and MIP-CuFe_2_O_4_/GQDs nanocomposite have hysteresis. The magnetic saturation values of CuFe_2_O_4_ nanoparticles and MIP-CuFe_2_O_4_/GQDs nanocomposite are 33.20 emu g^−1^ and 8.31 emu g^−1^, respectively. As can be seen, the magnetic saturation value of MIP-CuFe_2_O_4_/GQDs is very small compared to that of CuFe_2_O_4_ nanoparticles, which indicates the coverage surface of the CuFe_2_O_4_ with polymer, completely.

Figure 2a–c show SEM images of CuFe_2_O_4_ NPs, GQD, and MIP-CuFe_2_O_4_/GQDs, respectively. The SEM image of graphene quantum dot shows its fully layered structure. CuFe_2_O_4_ nanoparticles have a spherical shape with an average diameter of about 25.97 nm (Fig. 2d). In the case of MIP-CuFe_2_O_4_/GQDs, the imprinted layer surrounded the CuFe_2_O_4_ NPs, and the average particle size increased to about 255.87 nm (Fig. 2e).

**Figure 3:**
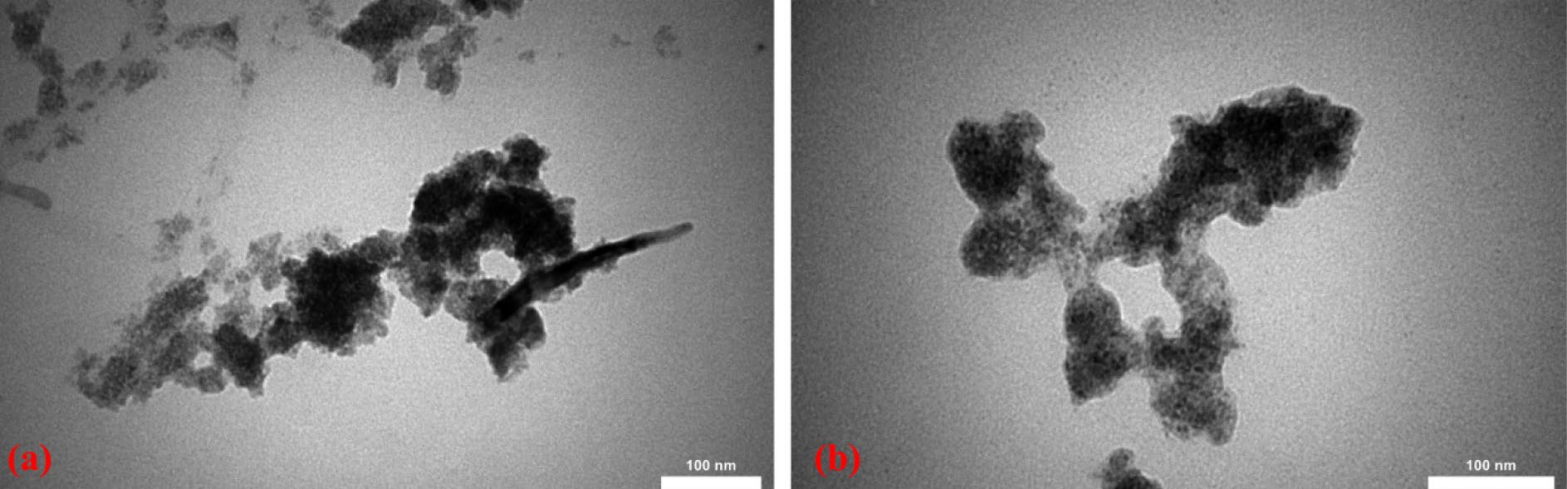
TEM image of (**a**) CuFe_2_O_4_ NPs, (**b**) MIP-CuFe_2_O_4_/GQDs nanocomposite.

The TEM image for CuFe_2_O_4_ NPs and MIP-CuFe_2_O_4_/GQDs nanocomposite are shown in Fig. 3. As can be seen in Fig. 3a, the CuFe_2_O_4_ NPs are clear and Fig. 3b shows CuFe_2_O_4_ NPs with a polymer layer on their surface. The SEM results are validated by the results of TEM.

### Results of RSM (CCD)

Experiments were prepared and conducted following the surface response technique and central composite design, and the results were evaluated using design expert software. Initially, the assumption of data normality was investigated using a normal distribution graph and a Box-Cox graph. According to the normal distribution diagram (Fig. S1-a)(see Supplementary Information (SI)), the data is distributed around the line and does not exhibit the "S" pattern, which confirming their assumption of normality^46^. The Box–Cox diagram (Fig. S1-b) (see SI) indicates that the best lambda value in the 95% confidence range of 0.12 to 1.49 is 0.77, and its current value is 1. Hence, it is within the range of confidence, and the data does not require a transfer function to be normalized. The modeling results reveal that the prediction model is a quadratic equation depending on the factors investigated. The developed model was statistically evaluated to identify the optimal quadratic model parameters for experimental data as a significant model using analysis of variance (ANOVA)^47^. As a consequence, based on the data acquired from the response prediction model and its variables, the final equation in terms of coded factors (a quadratic polynomial function) is as follows (Eq. (10)):

10 $$\begin{aligned} R\% &= 33.81039 + 13.46999{X_1} - 0.534203{X_2} - 4.16522{X_3} + 76.33413{X_4} \hfill \\ &\quad + 0.117479{X_1}{X_3} - 4.42371{X_1}{X_4} - 0.683844{X_1}^2 + 0.057012{X_2}^2 + 0.040231{X_3}^2 \hfill \\ \end{aligned}$$

The coded factors X_1_, X_2_, X_3_, and X_4_ in the above equation refer to pH, time, initial concentration of pirimicarb, and nanocomposite dosage, respectively. The ANOVA analysis results of the obtained quadratic model (Eq. (10)) are presented in Table 2. A significant model should have a non-significant lack-of-fit (p-value > 0.05) and a p-value less than 0.05^48,49^. A large F value and a low P value suggest the great significance of the related parameters^50^. As can be seen in Table 2, the F-value of 63.40 and corresponding p-value less than 0.0001 for the quadratic model confirm its significance. The non-significant lack-of-fit term (F-value = 2.63 and p-value > 0.05), implying that the residual error is not significant. Furthermore, summary statistics show R^2^, average value, SD, and CV% are equal to 0.96, 71.68, 3.87, and 5.40%, respectively. The obtained model is reproducible with regard to the coefficient of variation, which is less than 10%. Also, the predicted R^2^ of 0.90 and adjusted R^2^ of 0.95 are in good agreement, which demonstrate the model's incredible ability to predict experimental results. The AP statistic value was estimated to be 30.77, which represents the noise signal rate. About this value, a number higher than 4 indicates the model's appropriateness^51,52^.

**Table 2:** Analysis of variance (ANOVA).

Source	Sum of squares	df	Mean square	F-value	p-value	
Model	8559.88	9	951.10	63.40	< 0.0001	
X_1_-pH	1615.92	1	1615.92	107.71	< 0.0001	
X_2_-Time	829.99	1	829.99	55.32	< 0.0001	
X_3_-Cocentration	2071.23	1	2071.23	138.06	< 0.0001	
X_4_-Dosage	3087.41	1	3087.41	205.80	< 0.0001	
X_1_X_3_	88.33	1	88.33	5.89	0.0260	
X_1_X_4_	78.28	1	78.28	5.22	0.0347	
X_1_^2^	191.55	1	191.55	12.77	0.0022	
X_2_^2^	52.01	1	52.01	3.47	0.0790	
X_3_^2^	414.35	1	414.35	27.62	< 0.0001	
Residual	270.04	18	15.00	–	–	
Lack of Fit	250.93	15	16.73	2.63	0.2317	
Pure Error	19.11	3	6.37	–	–	
Cor Total	8829.92	27	–	–	–	

Model summary statistics						
Response	SD	C.V. %	R^2^	Adjusted R^2^	Predicted R^2^	AP
R%	3.87	5.40	0.9694	0.9541	0.9023	30.7764

Equation (10) displays the coefficients of all effective parameters. Since this equation does not provide enough information on the effect of each variable and its operation, the perturbation graph of independent variables was investigated (Fig. S2-a) (see SI). This graph indicates the impact of significant variables by demonstrating variations in the response of each factor as it moves away from the reference point (zero coded level of each factor), while all other factors remain constant at the reference point^53^. In the curve, the effect of the squared terms for all variables, except adsorbent dosage, can be observed. In addition, the slope of the graph related to time is lower in comparison to other factors, indicating that the response is less sensitive to this variable. In contrast, other variables have relatively similar slopes. Besides, increasing the initial concentration of pirimicarb decreases the adsorption process's effectiveness, whereas raising the other three influential factors leads to an increase in adsorption efficiency. Because, raising the initial concentration of pirimicarb causes its molecules to compete for placement on the polymer surface, which reduces adsorption efficiency^30^. On the Pareto chart (Fig. S2-b) (see SI), in addition to the importance of the effects of significant variables, their interactions can also be seen. As regards this chart, the polymer dosage (X_4_) has the most significant percentage of effectiveness, and time squared has the least percentage (X_2_^2^) in adsorption efficiency.

The three-dimensional graphs of the response surface of the adsorbed quantity of pirimicarb relative to the four factors were employed to get knowledge of variable interactions and evaluate the optimum amount of each variable to achieve maximum adsorption. Figure (S3-a) (see SI) depicts surface plots of pirimicarb adsorption percentage versus the pH and the polymer dosage by keeping contact time constant at 15 min and the initial concentration at 30 mg/L. In this graph, the maximum efficiency associated with the influence of two variables (pH and the polymer dosage) hit a peak at a pH of approximately 7 and the highest level of nanocomposite dosage (1250 mg/L). In Fig. (S3-b) (see SI), which demonstrates interactions between pH and the initial pirimicarb concentration in a constant time (15 min) and polymer dosage (750 mg/L), the maximum efficiency associated with the influence of these two variables reached its peak at a concentration of around 10 mg/L and the highest level of pH 7 to 11.

The parameters were optimized to obtain the conditions wherein all variables are ideal, and the adsorption percentage is highest. Figure S4 (see SI) depicts graphs of the actual quantity of each independent variable in which the optimal points are indicated, as well as graphs of the maximum and minimum adsorption percentage. The best parameters for removing 100 percent of pirimicarb are an initial pirimicarb concentration of 10.17 mg/L, a pH of 7.54, a contact time of 6.15 min, and a polymer dosage of 840 mg/L. This optimum condition was investigated experimentally, where 99.92 adsorption percent was attained, which has a slight discrepancy in comparison with the model estimated quantity. It can be inferred that the central composite design can efficiently characterize and estimate the optimal adsorption process circumstances for the pirimicarb by MIP-CuFe_2_O_4_/GQDs nanocomposite.

### Fluorescence performance and detection sensitivity and linear range of sensor

**Figure 4:**
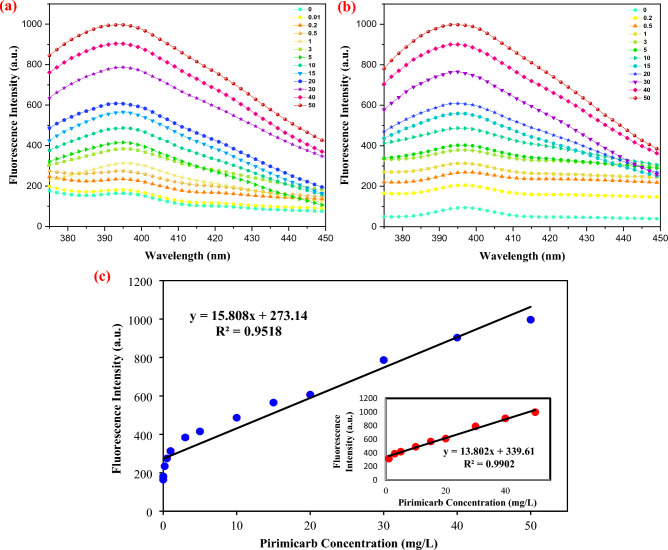
The results of fluorescence intensity with pirimicarb concentration: (**a**) all of the curves for experimental samples, (**b**) all of the curves for real samples, (**c**) calibration curve.

To evaluate the detection efficiency of the proposed sensor, specific dosages of the MIP-CuFe_2_O_4_/GQDs were separately dispersed in different concentrations of pirimicarb. Then the fluorescence emission spectra of these samples were obtained under the optimal experimental condition. Also, the river water samples, whose specifications are given in Table S3, was analyzed using this method, and the results are presented in Fig. 4b. For the evaluation of the sensor's detection sensitivity, the correlation of fluorescence intensity with pirimicarb concentration was conducted, and the results are provided in Fig. 4. As can be observed, the fluorescence signal gradually increase as the pirimicarb concentration is raised from 0 to 50 mg/L (Fig. 4a). The pirimicarb binding to polymer caused the turn-on fluorescent intensity that is generally related to the transition from π to π* of the C=C bonds and benzene rings or from n to π* of C=O bonds or others, respectively. The fluorescence intensity was increased via π–π interaction, and exhibits stable solid-state photo-luminescence (PL) to monitor the pirimicarb loading and releasing through the Förster resonance energy transfer with the pirimicarb like tramadol and doxorubicin^54–56^. The absorbance experiment showed that an interaction exists between pirimicarb and MIP polymer that turns on the absorbance behavior of MIP dependent on pirimicarb^57^. The fluorescence intensity of these samples at 395 nm are represented in Fig. 4c. The linear model of these data was calculated by Excel software (Eq. (11)). As can be seen, the R^2^ and sensitivity are equal to 0.9518 and 15.808 a.u.L/mg, respectively. The linear range can be measured by making a plot of analyte concentration versus fluorescence, and seeing at what concentration the data deviate from a straight line that is tangent to the low end of the concentration range. Therefore, the fluorescence intensity shows an excellent linear relationship with the pirimicarb concentration in 1 to 50 mg/L.

11 $$y = 15.808x + 273.14$$

The results of measurement of real samples (Fig. 4b) are reported in Table 3. Also, the relative standard deviations (RSD) of this measurement were calculated. As can be seen, the RSD of all data is less than 2, so real samples can be measured with acceptable accuracy by this chemosensor. The LOD and LOQ of this sensor were calculated by the calibration line at low concentrations. The results show that the LOD and LOQ are equal to 1.79 mg/L and 5.43 mg/L of pirimicarb concentration, respectively.

**Table 3:** Results of measurement of real samples and RSD.

Correct pesticide concentration	Measured value	Relative standard deviation
0.5	0	0.25
1	2.52	0.76
3	6.69	1.84
5	8.17	1.59
10	13.53	1.77
15	18.07	1.54
20	21.21	0.60
30	31.11	0.55
40	39.66	0.17

### Selectivity and binding sites

**Figure 5:**
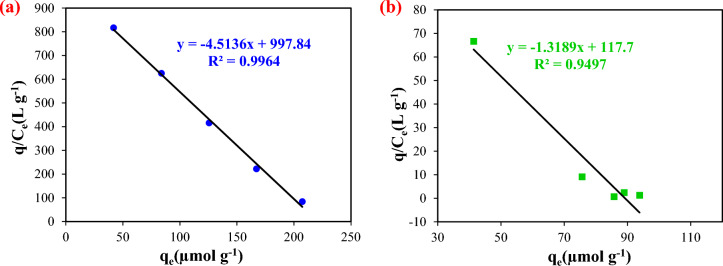
Scatchard plots of (**a**) MIP-CuFe_2_O_4_/GQDs nanocomposite, (**b**) NIP-CuFe_2_O_4_/GQDs nanocomposite.

To examine the selectivity of NIP-CuFe_2_O_4_/GQDs and MIP-CuFe_2_O_4_/GQDs nanocomposites, competitive adsorption of pirimicarb was performed separately with each of Diazinon, Deltamethrin, and Chlorpyrifos pesticides, and results are reported in Table S1 (see SI). According to the results, the NIP nanocomposite has a virtually amount of distribution coefficient (K_d_) for all intended pesticides. In contrast, the distribution coefficient of MIP nanocomposite to the adsorption of pirimicarb is considerably higher than that of the others. This reveals the high capability of selective pirimicarb adsorption by MIP nanocomposite, and a higher selectivity coefficient (K) for the MIP compared to the NIP nanocomposite. Besides, the relative selectivity coefficients of pirimicarb with each of the three other pesticides are roughly similar. In addition, this study was done for real samples and results of them are shown in Table S2. The relative selectivity coefficients of pirimicarb in real samples are similar to the results of laboratory samples. The Scatchard plot was also used to study the binding sites of MIP nanocomposite and compare it to NIP nanocomposite, and the results are shown in Fig. 5 and Table 4. As can be observed, the q_max_ coefficient for MIP nanocomposite is higher than that of NIP nanocomposite, and the imprinting factor value attained 2.48, which confirms the specific binding properties of pirimicarb pesticide. In Table 5, the comparison of the results of this study with other studies is reported. As can be seen, the chemosensor used in this study can compete with other similar chemical sensors. In addition, it has a much simpler structure than them. The detection limit value of the sensors reported in other studies is higher than the sensor developed in this study, but their working and linear ranges are also different. However, the maximum residue level of pirimicarb pesticide is equal to 3 ppm, which in this study; the developed sensor has the ability to detect much lower and higher amounts than. In addition, imprinting factor of MIP-CuFe_2_O_4_/GQDs is also suitable and comparable with other reported polymers.

**Table 4:** Results of Scatchard analysis.

Adsorbent	K_D_ (µmol/l)	q_max_ (µmol/g)	IF
MIP	0.221	221.07	2.48
NIP	1.32	117.7

**Table 5:** Comparison of the results with other studies.

Sensor material	Detection method	Analytes	Matrix	IF	Linear range	LOD	Reference
UCNPs@MIP	Fluorescence	Carbendazim	Food samples	2.21	0.01–1 μg/mL	0.0036 μg/mL	^58^
@MIH-prm	Fluorescence	Procymidone	Ginseng	3.01	1–40 nM	0.569 nM	^59^
N-GQDs/MIP	Fluorescence	Thiacloprid	Underground water	–	0.1–10 mg/L	0.03 mg/L	^60^
L‐cys‐CdSeTe/ZnS@MIP	Fluorescence	Atrazine	Tap and lake water	–	2–20 M	0.80 × 10^−7^ M	^61^
Fe_3_O_4_‐chitosan@MIP	Fluorescence	Atrazine	Water	–	2.32–185.4 µM	0.86 µM	^62^
MIP-QDs	Fluorescence	Propanil	Fish and seawater samples	1.4	1.0–20.0 × 10^3^ μg/L	0.6 μg/L	^63^
SiO_2_@Zn protoporphyrin – MIP	Fluorescence	Atrazine	Deionized water and lake water	–	0–1 × 10^−4^ M	1.8 µM	^64^
S-CQD	Fluorescence	Pirimicarb	Cereal samples	–	0.022–5 μg mL^−1^	0.006 μg mL^−1^	^65^
Flow Injection Analysis Assembly	Fluorescence	Pirimicarb	Pirimicarb in freshwater	–	4.25–30.75 ng mL^−1^	0.12 ng mL^−1^	^66^
MIP-CuFe_2_O_4_/GQDs	Fluorescence	Pirimicarb	Deionized water and river water	2.48	1–50 mg/L	1.79 mg/L or 7 μM	This work

### Reusability

For investigating the reusability of MIP-CuFe_2_O_4_/GQDs, in the first step, the best solution was selected (13 different pH of Britton–Robinson buffer). The results have been shown in Fig. S5-a (see SI). As can be seen, the best solution is the Britton–Robinson buffer at pH = 1. Then, up to 15 cycles of pirimicarb adsorption by the MIP-CuFe_2_O_4_/GQDs were carried out, and after each step, the polymer was washed by Britton–Robinson buffer (pH = 1). After each cycle, the percentage of adsorption was calculated, and the results are shown in Fig. S5-b (see SI). After 15 cycles, the adsorption percentage of MIP-CuFe_2_O_4_/GQDs for pirimicarb is higher than 96%. Therefore, MIP-CuFe_2_O_4_/GQDs have good reusability for pirimicarb pesticide as an optical chemosensor.

## Conclusion

In this study, the MIP-CuFe_2_O_4_/GQDs were synthesized and characterized by FT-IR, XRD, TEM, and SEM techniques. The results of this analysis showed the MIP-CuFe_2_O_4_/GQDs was correctly synthesized. Then, the ability of MIP-CuFe_2_O_4_/GQDs as an optical chemosensor was investigated. In the first step, the optimal condition for the adsorption of pirimicarb by this polymer was studied. The results showed the best conditions for maximum adsorption are pH = 7.54, initial pirimicarb concentration = 10.17 mg/L, polymer dosage = 840 mg/L, and contact time = 6.15 min. And in these conditions, the adsorption percentage is 99.92. The sensitivity, LOD, and LOQ for this optical sensor were calculated based on their fluorescence intensity, and are equal to 15.808 a.u.L/mg, 1.79 mg/L, and 5.43 mg/L, respectively. The selectivity and reusability of this sensor were studied, and the results demonstrated that the imprinting factor for MIP-CuFe_2_O_4_/GQDs is equal to 2.48. Reusability results of MIP-CuFe_2_O_4_/GQDs as a sensor shows that their adsorption percentage after 15 cycles is higher than 96%. Generally, according to the results of this study, the MIP-CuFe_2_O_4_/GQDs is the best chemosensor material for the detection of pirimicarb in an aqueous solution.

### Supplementary Information

Supplementary Information.

## Data Availability

The datasets used and/or analysed during the current study available from the corresponding author on reasonable request.
