# Supplementary material for: Development of a simple polymer-based sensor for detection of the Pirimicarb pesticide
Source: Sci Rep. 2024 May 4;14:10293. doi: 10.1038/s41598-024-60748-6 (PMC11069528; doi:10.1038/s41598-024-60748-6)
Supplement: Supplementary file 1 — Supplementary Information. [file 41598_2024_60748_MOESM1_ESM.docx]

Supplementary Information:

**Development of a Simple Polymer-based sensor for detection of the Pirimicarb pesticide**

**Zahra Saadatidizaji^a^, Negin Sohrabi^a,b^, Reza Mohammadi^a*^**

*^a^Polymer Research Laboratory, Department of Organic and Biochemistry, Faculty of Chemistry, University of Tabriz, Tabriz, Iran.*

*^b^Department of Biosystem Engineering, Faculty of Agriculture, University of Tabriz, Tabriz, Iran.*

[*r.mohammadi@tabrizu.ac.ir](mailto:*r.mohammadi@tabrizu.ac.ir)

**
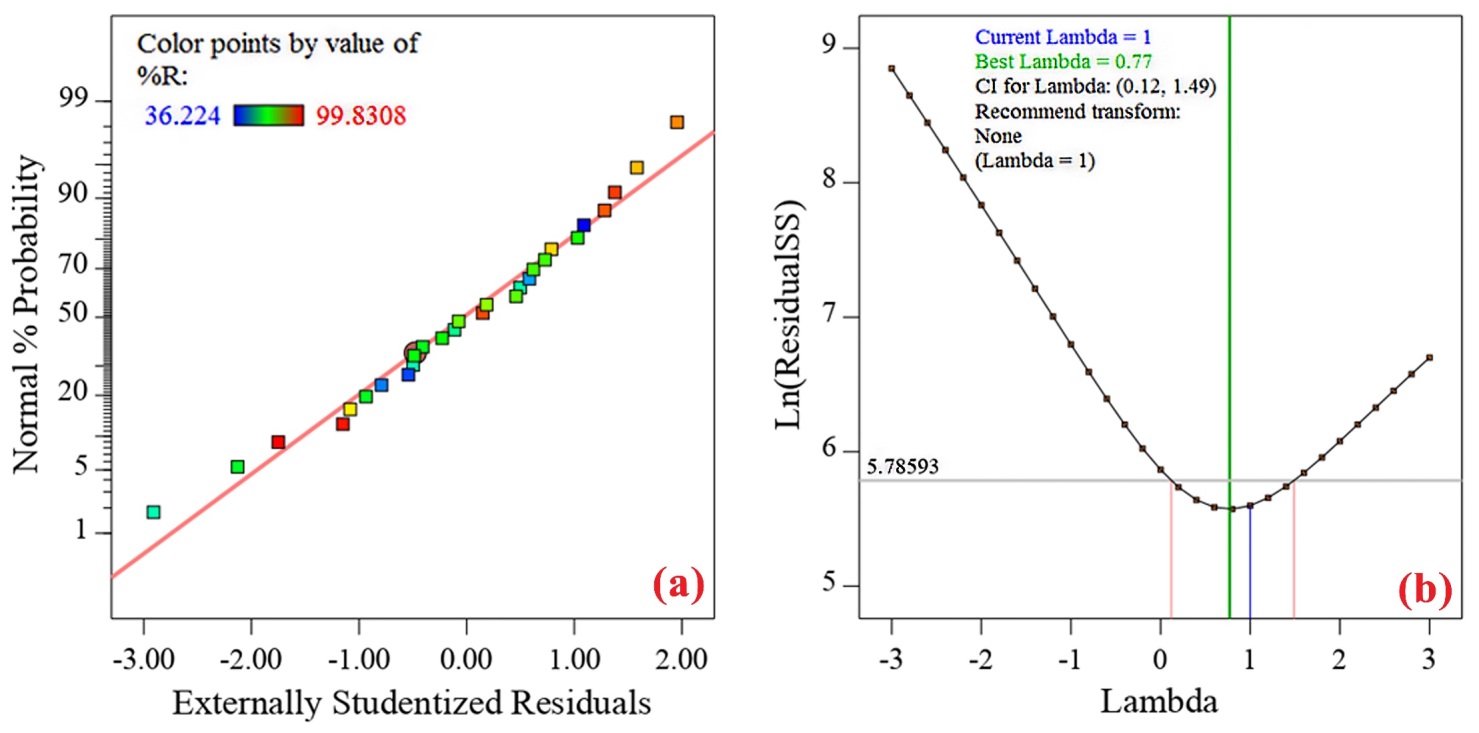
**

Fig S1. Response surface methodology results. a) Normal distribution graph, b) Box-Cox graph


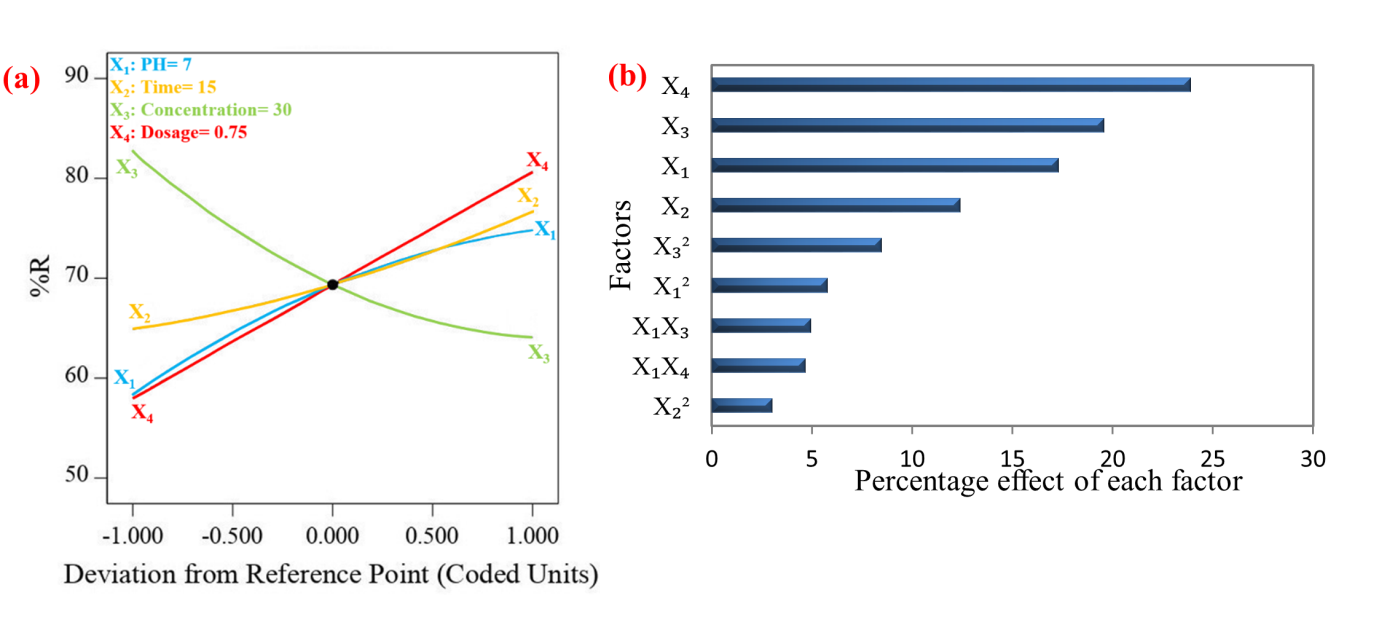


Fig S2. a) Perturbation graph of all factors in center point, b) Pareto chart for all major factors

**
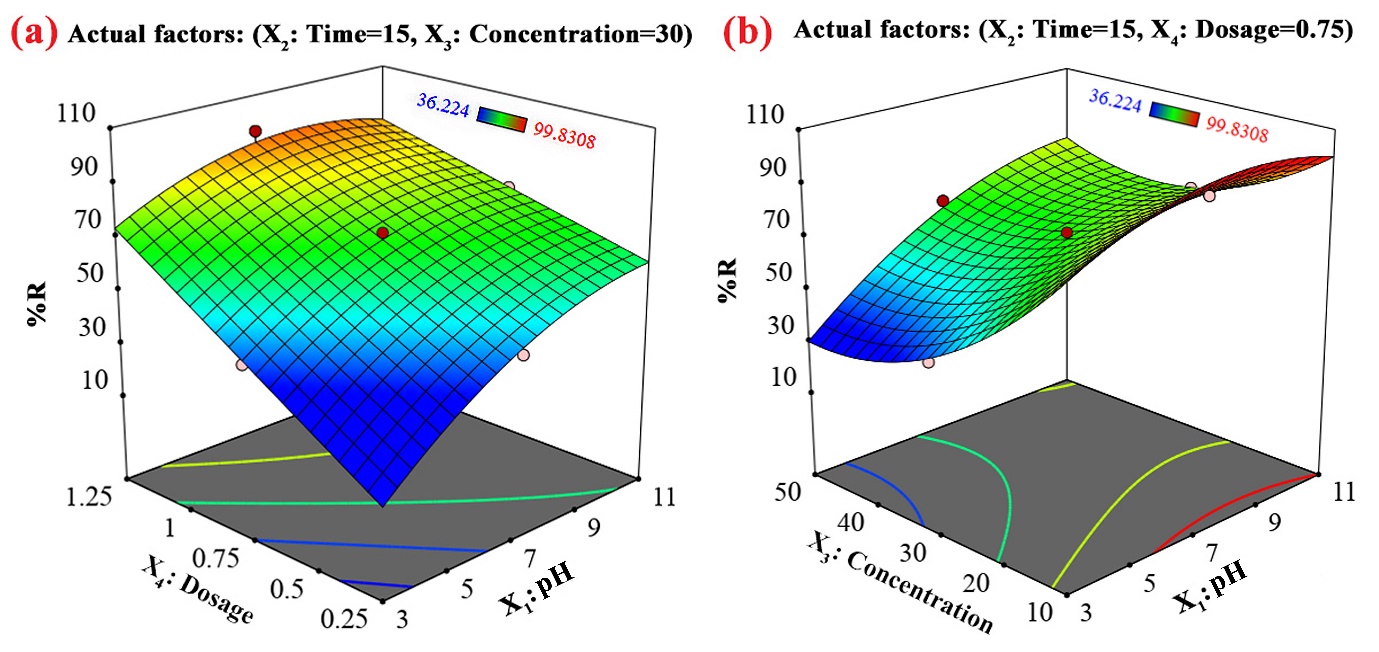
**

Fig S3. Three-dimensional graphs for interaction of a) pH and polymer dosage, b) pH and concentration of pirimicarb

**
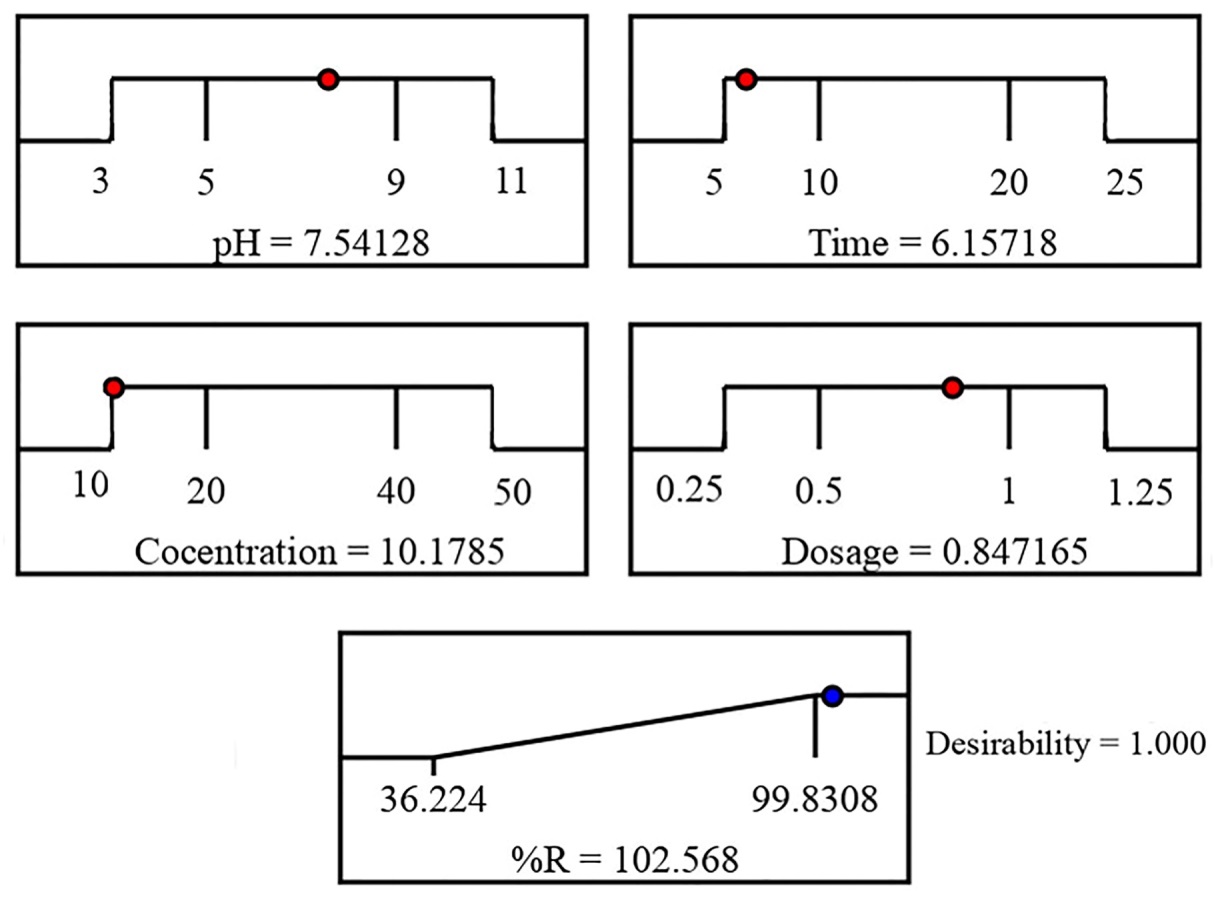
**

Fig S4. Actual profiles with predicted optimal parameters for pirimicarb adsorption by MIP-CuFe_2_O_4_/GQDs nanocomposite.

Table S1. The results of the selectivity study.

| K' | K | K_d_ (L/g) | | K' | | K | K_d_ (L/g) | | K' | K | K_d_ (L/g) | | adsorbent |
| --- | --- | --- | --- | --- | --- | --- | --- | --- | --- | --- | --- | --- | --- |
|  | | CPF | PRI | |  | | DLT | PRI |  | | DZN | PRI |  |
| 21.20 | 14.44  0.68 | 0.54  1.77 | 7.89  1.21 | | 19.63 | 17.95  0.91 | 0.5  1.14 | 8.97  1.04 | 21.42 | 27.81  1.29 | 0.33  1.33 | 9.22  1.72 | MIP  NIP |
| PRI: Pirimicarb, DZN: Diazinon, DLT: Deltamethrin, CPF: Chlorpyrifos | | | | | | | | | | | | | |

|  |  |
| --- | --- |

Fig S5. The plots of a) the desorption percentage in different pH levels of Britton–Robinson buffer for selecting the best solution, b) adsorption percentage in 15 cycle.

Table S2. RSM data

| Std | Run | pH | Time (min) | Concentration (mg/L) | Dosage (g/L) | R% |
| --- | --- | --- | --- | --- | --- | --- |
| 1 | 1 | 5 | 10 | 20 | 0.5 | 57.68 |
| 2 | 14 | 9 | 10 | 20 | 0.5 | 73.69 |
| 3 | 25 | 5 | 20 | 20 | 0.5 | 66.50 |
| 4 | 7 | 9 | 20 | 20 | 0.5 | 86.49 |
| 5 | 22 | 5 | 10 | 40 | 0.5 | 36.22 |
| 6 | 17 | 9 | 10 | 40 | 0.5 | 56.71 |
| 7 | 9 | 5 | 20 | 40 | 0.5 | 46.43 |
| 8 | 8 | 9 | 20 | 40 | 0.5 | 72.08 |
| 9 | 16 | 5 | 10 | 20 | 1 | 87.99 |
| 10 | 28 | 9 | 10 | 20 | 1 | 94.45 |
| 11 | 12 | 5 | 20 | 20 | 1 | 95.42 |
| 12 | 20 | 9 | 20 | 20 | 1 | 98.61 |
| 13 | 5 | 5 | 10 | 40 | 1 | 59.53 |
| 14 | 10 | 9 | 10 | 40 | 1 | 77.19 |
| 15 | 18 | 5 | 20 | 40 | 1 | 65.49 |
| 16 | 4 | 9 | 20 | 40 | 1 | 84.93 |
| 17 | 6 | 3 | 15 | 30 | 0.75 | 40.61 |
| 18 | 27 | 11 | 15 | 30 | 0.75 | 74.63 |
| 19 | 19 | 7 | 5 | 30 | 0.75 | 57.10 |
| 20 | 3 | 7 | 25 | 30 | 0.75 | 91.42 |
| 21 | 21 | 7 | 15 | 10 | 0.75 | 99.83 |
| 22 | 11 | 7 | 15 | 50 | 0.75 | 69.47 |
| 23 | 23 | 7 | 15 | 30 | 0.25 | 44.12 |
| 24 | 26 | 7 | 15 | 30 | 1.25 | 96.32 |
| 25 | 13 | 7 | 15 | 30 | 0.75 | 71.98 |
| 26 | 24 | 7 | 15 | 30 | 0.75 | 66.0167 |
| 27 | 2 | 7 | 15 | 30 | 0.75 | 67.5766 |
| 28 | 15 | 7 | 15 | 30 | 0.75 | 68.5237 |

Table S3. Characterizations of river water (COD) before and after adsorption process

| Sample | COD (mg O_2_/L) |
| --- | --- |
| River water | 198 |
| River Water with pirimicarb | 395 |
| After adsorption by CuFe_2_O_4_ as adsorbent | 50 |
| After adsorption by MIP-CuFe_2_O_4_ as adsorbent | 30 |
